# Diminished physical function in older HIV-infected adults in the Southeastern U.S. despite successful antiretroviral therapy

**DOI:** 10.1371/journal.pone.0179874

**Published:** 2017-06-29

**Authors:** Audrey L. Khoury, Miriam C. Morey, Tammy C. Wong, Donna Lynn McNeil, Barlett Humphries, Katherine Frankey, Carl F. Pieper, Charles B. Hicks, Kim Huffman, Mehri S. McKellar

**Affiliations:** 1 University of North Carolina at Chapel Hill School of Medicine, Chapel Hill, North Carolina, United States of America; 2 Claude D Pepper Older Americans Independence Center, Department of Medicine, Duke University School of Medicine, Durham, North Carolina, United States of America; 3 Geriatric Research, Education and Clinical Center, Veterans Affairs Medical Center, Durham, North Carolina, United States of America; 4 Office of Research Support, Duke Cancer Institute, Durham, North Carolina, United States of America; 5 Center for Applied Genomics and Precision Medicine, Duke University Medical Center, Durham, North Carolina, United States of America; 6 Duke Department of Biostatistics and Bioinformatics, Durham, North Carolina, United States of America; 7 Division of Infectious Diseases, University of California San Diego School of Medicine, San Diego, California, United States of America; 8 Duke Molecular Physiology Institute, Duke University School of Medicine, Durham, North Carolina, United States of America; 9 Division of Infectious Disease, Duke University Medical Center, Durham, North Carolina, United States of America; University of Texas Medical Branch at Galveston, UNITED STATES

## Abstract

**Background:**

As antiretroviral therapy efficacy improves, HIV is gradually being recognized more as a chronic disease within the aging HIV-infected population. While these individuals are surviving into old age, they may, however, be experiencing “accelerated aging” with greater declines in physical function than that observed among comparably matched individuals free of HIV. This decline is not well understood and it remains unclear if physical decline correlates with the degree of immunosuppression based on CD4 lymphocyte nadir.

**Methods:**

In a cross-sectional study of accelerated aging in the older HIV-infected population on antiretroviral therapy (ART), physical performance evaluations were completed on a cohort of 107 HIV-infected subjects, age 50 years or older (with no HIV-1 RNA >200 copies/mL in the prior 12 months), and compared to reference ranges for age- and gender-matched HIV-uninfected persons. Physical performance testing consisted of four validated assessments: the 2.4-meter walk, 30-second chair stand, grip strength and 6-minute walk test.

**Results:**

When compared to age- and gender-matched HIV-uninfected reference controls, older HIV-infected persons had diminished physical function. No correlation was found between physical function and degree of immunosuppression as determined by pre-ART CD4 nadir.

**Conclusions:**

Despite improved survival, HIV–infected adults on suppressive ART have diminished physical function compared to HIV-uninfected persons. The degree of HIV-associated immunosuppression does not correlate with the observed degree of physical function decline in older HIV-infected persons, suggesting the decline is mediated by other mechanisms.

## Introduction

Between 2007 and 2009, the prevalence of HIV-infected adults aged 50 years and older increased from 28.6% to 32.7% [1]. By the end of 2015, it has been estimated that greater than 50% of HIV-infected adults in the U.S. were age 50 years and older [2]. With long-term survival of HIV-infected persons now a reality, there is growing concern among patients and providers alike that older HIV-infected persons are experiencing ‘accelerated’ aging, including more rapid decline in physical function. Biological mechanisms for this apparent decline are poorly elucidated but are thought to include higher rates of co-morbidities and smoking, prolonged antiretroviral therapy (ART) [3], chronic HIV infection with concomitant inflammation and underlying immunosuppression [4], and/or low bone and muscle mass [5].

Prior studies of physical function among HIV-infected persons have mostly used participant self-report and have primarily included mostly younger male participants (averaging 50 years of age) [6–8]. In addition, previous studies describing functional decline have included participants with chronic pain, wasting syndrome, co-morbid conditions and/or symptomatic HIV infection–all of which can act as confounding variables to negatively impact physical capabilities [9–12]. For example, a cross-sectional analysis of self-reported data from the Veterans Aging Cohort Study was able to show that there was no significant difference in physical function between the average HIV-infected and HIV-uninfected 50-year-old. In addition, this study concluded that the presence of a co-morbid condition (chronic pulmonary disease) resulted in significant functional decline in HIV-infected adults relative to HIV-uninfected adults [6]. A cross-sectional analysis of physical function based on responses to a questionnaire demonstrated that higher CD4 counts and undetectable HIV-1 viral loads were associated with better physical function [13]. Finally, HIV-infected men in the Multicenter AIDS Cohort Study were found to have a greater-than-expected decline in accelerated longitudinal gait speed over time [14]. These aforementioned studies suggest diminished physical function in aging HIV-infected persons, but illustrate the lack of real-time, direct objective measurements of function in healthy older HIV-infected persons (both male and female) who have controlled HIV replication on ART.

To better assess physical function in older persons with HIV infection, we evaluated physical function using validated physical performance tests in older HIV-infected individuals on effective ART and compared these to published reference ranges for healthy controls of similar age and gender. We hypothesized that a history of significant immunosuppression (as assessed by CD4 nadir) would negatively impact current physical function.

## Methods

### Participants

HIV-infected subjects were recruited from the Duke University HIV Clinic in Durham, North Carolina, during 2013–2014. Eligibility criteria included: (a) age 50 years old or older at time of enrollment; and, (b) on suppressive ART for the past year (no HIV-1 RNA >200 copies/mL in the prior 12 months). Exclusion criteria included the presence of conditions that would prevent the subject from safely undergoing functional testing, such as: (a) active unstable angina, (b) recent (<6 months) history of myocardial infarction, (c) history of ventricular tachycardia, (d) uncontrolled hypertension (diastolic BP >120 mm/Hg), (e) recent (<6 months) history of stroke or any residual neurological deficits affecting physical function, (f) active substance abuse affecting participation in study, (g) dementia or diagnosis of mental or behavioral disorders that preclude study participation, (h) severe hearing or vision loss, and (i) chronic pain that may limit performance testing. Subjects completed a one-time visit to capture the following: demographics, body mass index (BMI), estimated duration of HIV infection, prior/current ART history, HIV-associated opportunistic infections, co-morbidities, lifestyle habits including tobacco and alcohol use, nadir/current CD4 counts, and initial HIV-1 RNA. All physical performance tests were performed during this same visit.

The protocol was approved by the Duke University Institutional Review Board (IRB# Pro00032905), and all participants gave written informed consent.

### Physical performance testing

Participants underwent physical performance testing using four validated functional assessments: the 2.4-meter walk, 30-second chair stand, grip strength, and 6-minute walk test. All tests were performed by trained study staff according to standardized protocols.

The 2.4-meter walk test is designed to measure gait speed (measured in meters per second), walked on a measured course at usual and maximal walking speed. Participants were given several meters to accelerate and decelerate before and after the test distance. This test is an effective indicator of functional status and is predictive of future institutionalization and survival/mortality [15]. The 30-second chair stand, which assesses lower extremity strength, requires subjects to stand all the way up and down from a chair as many times as possible in 30 seconds. This test can discriminate between fallers and non-fallers [16]. Grip strength was measured via a Jamar dynamometer, an instrument that tests hand function and/or strength. Subjects squeeze the dynamometer which then records the intensity of grip strength. The grip strength test has been found in other studies to be predictive of mortality [17]. Aerobic endurance is measured by the 6-minute walk test. Subjects walk as fast as possible down a hallway and around a cone and back for 6 minutes. The 6-minute walk test is a good indicator of aerobic capacity [18].

### Reference controls

Physical performance test results for this study were compared to well-established reference ranges for healthy controls of similar age and gender from the literature [15–21]. Additionally, differences for gait speed and the 6-minute walk test were calculated between our observed values and the age- and gender-matched reference ranges and were compared to clinically significant meaningful differences published in the literature (available for gait speed, 30-second chair stand, and 6-minute walk test) [22–23].

### Comorbidity index

A co-morbidity index (ranging from 0–5) was created by summing the number of co-morbidities each participant reported, including cardiovascular, pulmonary, renal, liver, and malignancy. Each participant was thus assigned an index score, which was subsequently used in statistical analysis.

### Statistical analysis

Statistical analyses were performed using SAS software, version 9.4 (SAS Institute, Cary NC) [24]. Demographic statistics are displayed by frequency and percentages for binary and ordinal variables, and by means and standard deviations for continuous variables. For predictor group comparisons, by, for example, CD4 nadir groups, the mean of the results of each physical performance test was calculated and shown. To control for non-normal distributions in the outcomes, differences between groups were tested using a 2-sample Wilcoxon or Kruskal-Wallis tests. Pearson correlations were used to test the impact of various antiretrovirals (ARVs) on physical function, partialling for 11 pre-selected confounding variables (BMI, gender, race, age, estimated duration of HIV, CD4 nadir, current CD4 count, tobacco use, alcohol use, illicit drug use, and co-morbidity index).

## Results

A total of 107 older HIV-infected persons on suppressive ART (HIV-1 RNA <200 copies/mL in the prior 12 months) were enrolled. Of these, 75 (70%) were male (Table 1) with a mean age of 60.3 years (range 50.2–78.1 years) and mean BMI of 28.6 kg/m^2^ (range 17.4–51.2 kg/m^2^). Most participants were Black/African American (n = 64; 60%) or white, non-Hispanic (n = 40; 37%). The mean duration of known HIV infection was 15.3 years (range 1.4–31.7 years), and the most common current ART regimen was efavirenz/emtricitabine/tenofovir disoproxil fumarate (n = 30, 28%).

**Table 1:** Overall demographics and clinical characteristics.

		Total (n = 107)	CD4 nadir <200 cells/mm^3^ (n = 73)	CD4 nadir >350 cells/mm^3^ (n = 34)
**Age**	50–59 years old	52 (49%)	37 (51%)	15 (44%)
	60–69 years old	49 (46%)	31 (42%)	18 (53%)
	≥70 years old	6 (6%)	5 (7%)	1 (3%)
**Gender**	Male	75 (70%)	50 (68%)	25 (74%)
	Female	32 (30%)	23 (32%)	9 (26%)
**Race/Ethnicity**	Black/African American	64 (60%)	48 (66%)	16 (46%)
	White/Caucasian	40 (37%)	23 (31%)	17 (50%)
	Hispanic	3 (3%)	2 (3%)	1 (3%)
**BMI**	< 25 kg/m^2^	28 (26%)	21 (29%)	7 (21%)
	25–29.9 kg/m^2^	42 (39%)	30 (41%)	12 (35%)
	> 30 kg/m^2^	37 (35%)	22 (30%)	15 (44%)

BMI = Body mass index.

At enrollment, the mean CD4 count of the cohort was 691 cells/mm^3^ (range 83–2,096 cells/mm^3^). The majority of subjects (n = 73; 68%) had previously had a nadir CD4 count <200 cells/mm^3^, and 42/107 (39%) had had a CD4 nadir <50 cells/mm^3^. Demographic and clinical characteristics were similar between the low and higher CD4 nadir groups (Table 1) with a trend towards significance for race by CD4 nadir. A total of 58 (54%) of the cohort had experienced HIV-associated opportunistic infections, most commonly *Pneumocystis* pneumonia (n = 23; 21%) and esophageal candidiasis (n = 36; 34%).

Overall, the sample had a modest number of co-morbidities with the mean co-morbidity index being 1.44. The most common co-morbidities were cardiovascular disease (61 participants [57%]), liver disease (39 participants [36%]), renal disease (18 participants [17%]), pulmonary disease (18 participants [17%]), and malignancy (17 participants [16%]). Of the 73 participants (68%) who were current or former smokers, the mean cumulative smoking history was 25.6 pack-years.

There was no significant effect on physical function from any particular type of antiretroviral therapy, including the use of drugs with greater potential for mitochondrial toxicity (didanosine, stavudine, and zidovudine) [25], even after controlling for a range of variables (including BMI, gender, race, age, estimated duration of HIV, CD4 nadir, current CD4 count, tobacco use, alcohol use, illicit drug use, and the co-morbidity index).

Individually, some of the demographic variables were related to physical function (Table 2). Most notably, males performed significantly better on grip strength and gait speed (measured by 2.4-meter walk at both usual and maximal speed). Participants with lower BMIs performed better on the 2.4-meter walk at both usual and maximal speed. Compared to blacks, white participants performed better on the 2.4-meter walk at both usual and maximal speed. Increasing age and higher co-morbidity indices both correlated with worse performance on the 6-minute walk test. Participants who were not current smokers had significantly better performance on the 30-second chair stand and the 6-minute walk test. Alcohol and illicit drug use, HIV duration, CD4 nadir, and current CD4 count did not significantly affect physical function. Additional analyses were performed to control for type-1 error in which age, gender and the co-morbidity index were found to be the most related to the outcomes of interest. While there was indication that other variables were related (such as white race and HIV duration), the multivariate P value, when controlling for other predictors, was not significant.

**Table 2:** Correlation between clinical characteristics and physical function.

	Gait speed–usual (m/s)	Gait speed–max (m/s)	30-second chair stand	Grip strength (kg)	6– minute walk test (m)	Multivariate p value, individual predictor	Multivariate p value, controlled for all other predictors
**Age**	-0.16	-0.17	0.011	-0.16	-0.28**	0.0099	0.0500
**Male gender**	0.23*	0.25**	0.17	0.65***	0.18	<.0001	<.0001
**White race**	0.25**	0.33***	0.18	0.12	0.17	0.0272	0.1505
**BMI**	-0.22*	-0.25**	-0.15	-0.078	-0.14	0.1717	0.3150
**Current tobacco use**	-0.16	-0.11	-0.24*	-0.046	-0.22*	0.1493	0.2306
**Co-morbidity index**	-0.14	-0.094	-0.12	0.086	-0.43***	<.0001	0.0004
**Current alcohol use**	0.16	0.15	0.11	0.13	0.15	0.6282	0.9986
**Current illicit drug use**	0.0047	0.017	0.0014	0.074	-0.15	0.2555	0.4621
**HIV duration**	0.011	0.15	-0.11	-0.053	-0.10	0.0188	0.3215
**CD4 nadir**	0.060	-0.039	-0.024	-0.021	-0.023	0.8095	0.6749
**Current CD4 count**	0.064	0.043	-0.0079	-0.050	0.063	0.8978	0.8462

All values listed are Pearson Correlation Coefficients. N = 107. Prob > [r] under H0.

*p<0.05;

**p<0.01;

***p<0.001

Gait speed was measured by the 2.4-meter walk test.

In our study, HIV-infected patients had diminished physical performance when compared to gender- and age-matched reference standards obtained from the literature [15–21] (Table 3). Additionally, there was a clinically significant, meaningful difference of >0.1 m/s for gait speed and >50 m for the 6-minute walk test [22–23] (Table 4).

**Table 3:** Physical function compared to normal reference values ranges.

	Gait Speed–Usual Speed (m/s)	Normal Values Usual Speed (m/s)	Gait Speed–Maximal Speed (m/s)	Normal Values Maximal Speed (m/s)	30-Second Chair Stand	Normal Values	Grip Strength (kg)	Normal Values (kg)	6-Minute Walk Test Distance (m)	Normal Values (m)
**Total (n = 107)**	1.17		1.76		12.72		33.45		432.99	
**Female age 50–59**	1.14	1.40	1.65	2.00	11.38	N/A	24.09	29.9–30.9	421.75	N/A
**Female age ≥60**	1.02	1.27–1.30	1.58	1.74–1.77	11.88	10–17	23.81	18.0–25.9	388	471–538
**Male age 50–59**	1.23	1.39	1.85	2.07	13.19	N/A	39.04	44.1–50.6	479.36	N/A
**Male age ≥60**	1.19	1.33–1.36	1.80	1.93–2.08	13.18	11–19	36.09	28.0–41.7	414.45	527–572
**CD4 nadir <200 cells/mm**^**3**^	1.15	N/A	1.76	N/A	12.66	N/A	33.19	N/A	427.40	N/A
**CD4 nadir >350 cells/mm**^**3**^	1.21	N/A	1.77	N/A	12.85	N/A	34.01	N/A	444.83	N/A

Values reported as *means*.

Gait speed was measured by the 2.4-meter walk test.

Normal values were obtained from the literature using established reference standards appropriate for age and gender-matched HIV-uninfected persons [15–21].

**Table 4:** Differences in physical function between HIV-infected study participants and normal reference ranges (controlled for age and gender).

	n	Difference from Norms	Clinically Significant Meaningful Difference
**Gait Speed–Usual Speed (m/s)**	107	-0.19 ± 0.25	>0.1 m/s
**Gait Speed–Maximal Speed (m/s)**	107	-0.21 ± 0.37	>0.1 m/s
**30-Second Chair Stand**	55*	-1.11 ± 4.06	2.0–2.6
**Grip Strength (kg)**	107	-3.68 ± 8.65	Not established
**6-Minute Walk Test Distance (m)**	106**	-105.15 ± 100.85	>50 m

*For the 30-second chair stand, 52 participants were removed from the analysis as age/gender-matched references were unavailable for persons <age 60.

**One person did not perform the 6-Minute Walk Test.

Gait speed was measured by the 2.4-meter walk test.

Clinically significant meaningful differences were obtained from the literature [22–23].

### Impact of CD4 nadir on physical function

Physical function did not significantly differ between those with a CD4 nadir of <200 versus >350 cells/mm^3^ (Table 3). There was also no significant difference in physical function between those with a CD4 nadir of <50 versus >350 cells/mm^3^.

## Discussion

The number of HIV-infected older adults is increasing in clinics worldwide, and ensuring optimal care for this population is an important priority. In this study, HIV-infected older persons on virologically suppressive antiretroviral therapy demonstrated significantly diminished physical function compared to similarly aged persons without HIV infection, putting them at risk for falls and mobility-related disability [26–28].

Using established reference standards appropriate for age and gender-matched HIV-uninfected persons [15–21], physical function was diminished in all performance measures, most notably in gait speed and the 6-minute walk test. These results may in fact predict these HIV-infected persons will not be physically independent at age 90 [19]. For example, a 60-year-old male should be able to do 17 chair stands in 30 seconds and walk 680 yards (622 m) in 6 minutes to be independent at age 90. In our cohort, males under the age of 60 were only able to do an average of 13.19 chair stands in 30 seconds and walk 479.36 m in 6 minutes.

There was a clinically significant, meaningful difference of >0.1 m/s for gait speed and >50 m for the 6-minute walk test between our subjects and the reference standards [22–23]. Based on our findings, the average walking speed for older HIV-infected subjects was slower than what is needed to safely cross the street (1.2 m/s) [29]. The difference for the 6-minute walk test was especially large at -105.15 m, more than two times the clinically significant meaningful difference of 50 m.

Possible reasons for diminished physical function include HIV infection itself, ART-related adverse events/toxicities, and/or the presence of significant co-morbidities. Although older nucleoside reverse-transcriptase inhibitors (NRTIs) such as didanosine, stavudine, and zidovudine [25] have been shown to cause mitochondrial toxicity, a history of having taken these medications did not significantly affect physical function in our cohort, even after controlling for confounding variables. Individually, variables such as male gender, lower BMI, current non-smoker status, white race, younger age, and lower co-morbidity index were associated with better performance on certain physical function tests. Since BMI and smoking are modifiable factors, they are potential targets for future interventions.

It should be noted that physical function did not significantly differ between those with a history of greater immunosuppression (CD4 nadir <50 or <200 cells/mm^3^) and those with lesser immunosuppression (CD4 nadir >350 cells/mm^3^). The relatively small sample size precludes definitive conclusions, but these data suggest that while the observed decline in physical function is clearly related to HIV infection, it is not mediated by the degree of prior immunosuppression experienced prior to initiation of ART.

This study has a few geographical and demographic limitations; our cohort was based in the Southeast and predominantly black. While it is unclear if our results are specific to the Southeast, this region carries a heavy burden of the U.S. HIV epidemic. Despite only comprising a third of the overall U.S. population, the Southeast accounts for an estimated 44% of all people living with HIV [30]. With regards to new infections, North Carolina is ranked in the top 10 states, with Durham County ranking third of all 100 counties [31]. In addition, although the U.S. Census Bureau projections for July 2015 noted that the population aged 50–69 is approximately 80% white and 5% black, blacks represent 37.2% of Durham County’s HIV-positive population compared to 13% of the overall U.S. population [32]. Therefore, our cohort was representative of the HIV-positive patient population in Durham County, North Carolina.

The magnitude of differences observed between our study participants and healthy reference controls appears to predict poorer health status, greater disability, increased medical-surgical visits, longer hospital stays, and higher health care-associated costs in older HIV-infected patients. This emphasizes the importance of finding appropriate intervention strategies and warrants additional research regarding mechanisms and predictors of decline. The implications of these data are significant for planning long-term HIV management approaches, particularly strategies for preserving physical function and quality of life in older HIV-infected patient populations.
